# Amputation for Scrofulous Diseases of the Joints

**Published:** 1846-06

**Authors:** Daniel Brainard

**Affiliations:** Professor of Surgery in the Rush Medical College


ARTICLE II.

*Amputations for Scrofulous Diseases of the Joints.* By Daniel Brainard, M. D., Professor of Surgery in the Rush Medical College.

Among the most certain and cheering signs of the progress of Surgery, is the fact that many diseases, supposed formerly to require operations for their cure, are now treated with equal success without a resort to the knife.

Scrofulous disease of the larger articulations is among this number. It is but a few years since, nearly every case of it was supposed to require amputation, and surgeons were enjoined to resort to it early, in order to prevent the effects of extensive suppuration. More recently this rule was modified and we were directed to wait until, from the effects of disease, the life of the patient was in imminent danger, when suppuration, emaciation, diarrhœa, and night sweats, were supposed to fortel impending dissolution. Quite lately it has been questioned whether, even under these circumstances, recovery without an operation might not take place; and the advocates of this opinion are at present numerous, and are yearly increasing. Convinced that this is the case, and knowing that limbs are still occasionally sacrificed, even before suppuration has taken place, we propose here to detail some cases where recovery without amputation has taken place, under circumstances, where from the established rules of practice, this might have been supposed to be impossible.

The following extracts from standard authors, may be considered as expressing the general opinion of surgeons at the present time.

"When the disease has got into this state, the constant pain, irritation, and discharge, bring on hectic symptoms of the most destructive kind, such as total loss of appetite, rest and strength, profuse night sweats, and as profuse purging, which foil all the efforts of medicine, and bring the patient to the brink of destruction." "It is an incontestible truth, that patients thus situated must perish, and it is equally true, that numbers in the same circumstances, by submitting to the operation, have recovered vigorous health."—*Cooper's Surgical Dict.* Vol. I. Art. Amputation.

"If, then, the articular capsule is fungus or full of pus, if fistulous ulcers transmit externally fetid pus, if the bones, cartilages, and tendons are changed, if the bones are separated, if there is great pain, loss of sleep, night sweats, hectic fever and diarrhea, conducting the patient to the brink of the tomb, amputation is formally indicated."—*Dict de Med.* Vol. II. Art. Amputation.

There are many, indeed the greater number of surgeons, who do not wait for all these symptoms, and yet it is probable that of those affected with them, as great a number recover without amputation as with it.

The following cases will show the foundation of such an opinion.

Case I.—*Inflammation of the knee, of three months standing,*

*caries of the bones and suppuration; recovery without amputation.*—Feb. 21, 1838, I was called to visit J. B., a boy of 12 years of age, of scrofulous habit, affected with inflammation of the right knee. The history of the case, as far as ascertained, was as follows: About three months previously he had a fall, and injured the joint slightly. This was soon followed by slight pain, gradual enlargement and heat, which, increasing, gave rise to constitutional irritation. He was treated with antiphlogistic remedies, but the disease of the joint progressed until the period when I saw him, when it presented the following appearances. The entire limb was much swollen from the toes to the hip, and about the knee there was redness and exquisite tenderness. Several points presented distinct fluctuation, which extended to the popliteal space, downward upon the sides of the leg, and upward to near the hip.

There was great emaciation, dry tongue, frequent pulse, chills and profuse night sweats, with diarrhea.

Free openings were made about the joint, which gave exit to an immense quantity of thin, whey-like pus, and on passing a probe, the articulur surfaces of the tibia and femur were found rough and carious. The patient was put upon the use of acids, bitter tonics, and anodynes to allay pain, but for two weeks no improvement took place. Amputation was then proposed, but the terror it excited in the patient, and the great aversion of the parents, prevented its acceptance. He was accordingly continued upon a tonic course, and as the serous discharge from the joint and the adjoining purulent foci, was abundant and offensive, these were freely injected with a solution of sul. copper, gr. jv to the oz. of water. For two weeks longer, scarcely any change could be perceived, but at the end of this time a diminution and improvement in the quality of the discharge was noticed. Soon after, the great tenderness having been diminished, a many tailed bandage was applied from the ankle to the hip, so as to remove the edema, and compress the purulent cavities.

As soon as the stomach could retain it, rich beef soup was given. Under this course of treatment there was a gradual improvement, and at the end of June—more than four months from the commencement of the treatment—he was able to walk on crutches,—the knee being anchylosed in the straight position. With the exception of this anchylosis, entire recovery took place, and the young man is at the present time (1846) able to follow a laborious occupation.

Case II.—*Scrofulous disease of the ankle of six years standing, suppuration and caries; recovery without amputation.*—Hogan, aged about thirty years. This was a case of scrofulus disease of the ankle, of six years standing, which came under treatment in the dispensary of the Medical College at Chicago, in the winters of 1843, '44, and '44, '45. Suppuration had continued for a long time, but at length ceased. The limb was emaciated, the joint enlarged,—stiff and cold, and the member quite useless. The general health was much impaired, but there were no symptoms indicating danger to his life. He had been an inmate of many hospitals, and amputation had been advised, which he declined from timidity. He was put upon a good diet, with hyd. potass, gr. x, twice daily. This was continued at intervals, for several months. A firm immovable apparatus of starched cotton rollers, was applied so as to effect the following objects, viz: preserve perfect immobility of the ankle, gently compass it and preserve its temperature. This treatment was persevered in eighteen months, at the end of which time, only a stiffness and rigidity —the effect of the disease—remained, and in March, 1846, he was in good health, using the limb freely, and pursuing an active employment.

Case III.—*Chronic Scrofulous disease of the knee, of long standing, suppuration; recovery without amputation.*—July 6, 1843, prescribed for F. M., a girl of 12 years of age, affected for many years with an enlargement of the inferior extremity of the right femur, attended with flexion of the leg to an angle of 45° with the axis of that bone. There was pain, slight and occasional, heat moderate, synovial effusion into the joint considerable, with impaired digestion, and an irritable debilitated state of the constitution. She was put upon a course of tonics with good diet, free exercise in the open air, while the knee was preserved in a state of perfect immobility, and protected from changes of temperature.

For upwards of a year the state of the disease, and the general health improved, so that she commenced to use the limb. This was followed by a return of the heat, pain, and swelling, in a greater degree than before, and notwithstanding that these were combatted by repose and antiphlogistic treatment, extensive suppuration took place, and a free opening was made with the caustic potash, upon the outside of the joint, Feb. 1, 1845.

Free suppuration, with all its local and constitutional effects, was established, and the same treatment adopted as in the first case, and at the end of five months from the time of making the opening, it was entirely healed, the general health good, and only a false anchylosis, in a partially flexed position remaining. By the use of gently extending means, this is so much removed, that she walks without difficulty, her general health is excellent, and but a few weeks more will be required to entirely straighten the joint.

Case IV.—*Caries of the Ankle. Long continued suppuration with hectic fever. Amputation and recovery. Return of the disease in the form of tuburcular consumption. Death.*—This case being one in which we were only called occasionally to consult, we were only acquainted with the most prominent facts and not with the details.

It was first seen July 13, 1841, and presented at that time the usual appearances of that articulation when affected with long continued caries and suppuration.

There was also hectic fever with its usual attendants, emaciation, diarrhea, &c. Amputation was performed by the attending surgeon, on the 18th of August following; the stump healed well, and the patient soon recovered his usual *embonpoint* and health. Symptoms of phthisis, however, soon developed themselves, and when last visited by us in February, 1843, he was in the last stage of that disease, and died soon after.

Cases like the last are unfortunately extremely common; so much so, that we doubt whether there is any surgeon in considerable practice, whose experience would not furnish several cases of the same kind. Instead of reporting them as we could from our own practice, we prefer to select some from the experience of others. The following are from Brodie on the diseases of the Joints, page 214. "A girl was admitted into St. George's hospital, who labored under this disease in the bones and joints of the tarsus. The foot was amputated by Mr. Griffiths. In about three weeks the stump was perfectly healed; but now she was seized with symptoms which indicated an affection of the mesenteric glands, which had not shown itself previously, and she died. On dissection, numerous glands of the mesentery were found enlarged, and containing cheesy matter. Another girl whose arm I amputated on account of a scrofulous disease of the elbow, became affected in the same manner immediately after the stump was healed. She also died, and similar appearances presented themselves on dissection. A man whose leg was amputated on account of a scrofulous disease of the tursus, in a short time after the operation, began to experience symptoms, which indicated the incipient state of some pulmonic complaint, and soon afterwards, the other foot was affected in the same manner as the first. These are a few of many cases which might be adduced as lending to the conclusion — that the occurrence of this scrofulous disease in a particular joint, may be the means of preventing the scrofulous disposition from showing itself in some other organ; and that if the affected joint be removed by an operation, there is more danger of the disease breaking out elsewhere, than there would have been if the operation had not been resorted to.

Druitt, in his Surgery, (p. 267) expresses the same opinion and even carries it further. "It seems probable that disease of the lungs or mesentery, is sometimes suspended or *averted* by the continuance of a (not very severe) disease of the extremity."

Numerous opinions of the same kind might be added, but we have lately met with the following passage in the work of R. W. Tamplin on deformities, which so well accords with our own experience, that we give it entire in place of multiplying authorities on the subject.

"A case occurred to me three or four years since, of which the following was the condition of the patient:—The girl, about 8 years of age, was sitting on the bed in the most miserably emaciated condition, and was stated to have been suffering from disease in the knee between two and three years. There were five openings, two on the outside, just above the condyles, one on the inside, and two on the patella. The three first communicated with the femur, the two last with the patella. The parents stated that at least half a pint of matter was discharged daily. Of course, this was somewhat exaggerated, but an immense discharge was then evident, of that peculiar, thin, unhealthy secretion, common in scrofulous diseases. The knee was contracted beyond a right angle, so that you could not pass a thin piece of sponge between the leg and thigh, in the situation of the popliteal space. The knee itself was swollen, and distended with fluid. She was suffering from acute hectic fever, with profuse perspiration and severe pain on the slightest motion. The pulse was fluttering, the bowels relaxed; in fact, the child presented the appearance of a person in the last stage of consumption. I ordered her an opiate, with the hyd. c. creta, every night, the conf. aromat. and ext. cinch. three or four times a day, a soft linseed-meal poultice, made by stirring the meal into boiling water, over the whole of the knee joint, covered with oiled silk. A tin splint, bent at the angle at which the knee was flexed, with pads, and retained by means of a flannel bandage, from the toes upwards above the knee, thereby keeping up the natural temperature of the entire limb, as well as a uniform gentle support; together with a nourishing diet, consisting of eggs, milk, meat, beer, and then two glasses of port wine daily, commencing the stimulants by degrees. In a few days the general irritability subsided, and the pain in the knee was relieved, the hectic left her, and the discharge altered its character. In fourteen days she could be moved without a complaint.

"This treatment was continued six months, varying the tonic at the end of which time all the openings were closed, and the swelling of the joint almost gone. The leg was extended in the most insensible manner, by gradually straightening the splint during the period the disease was subsiding, and without pain being produced. As soon as the openings were healed, I supported the joint with emp. cerat. saponis, and continued the bandage and splint, and in twelve months the leg was brought into the straight position, and the girl could use it without any assistance. I then directed the joint to be exercised daily, by forcibly flexing and extending it, as far as the feelings of the child would admit, and in this way the motion of the joint was restored, the muscles of the thigh and calf developed themselves, and a perfectly useful limb is the result. In this case amputation was advised by several surgeons as the only means of saving the child's life.

"I would beg to mention another case, similar in some respects, of a boy 9 years of age, in whom what is called white-swelling had existed for eighteen months. There were no openings, but a bag of matter situated in the upper and outer side of the femur just above the condyles. The joint also distended with fluid, and contracted beyond a right angle. The same treatment was followed here; the matter gradually and entirely disappeared, the swelling of the joint reduced, and by means of the splint the leg was brought, in eight months, into the straight position, and the boy enabled to use his leg, supported with a straight splint. In this case also amputation was recommended, and would certainly have been carried into effect, had the boy assented. I have used the poultice made in the manner described, as it acts both as a fomentation, and enables you also to apply slight support to the distended and weakened capillaries, over the whole surface. The splint keeps the joint steady, and relieves the pain, at the same time that a permanent contraction is prevented by the gradual and steady extension kept up, and the flannel bandage maintains the temperature and gives a uniform support; for it is certain that no restorative process can go on if the natural temperature is reduced, which you will frequently find to be the case in these subjects. The opium greatly allays the general irritation from which they suffer so severely in consequence of continued pain, and is, according to my experience, of the greatest possible advantage.

"I have ventured on this digression, because I believe that many legs are lost simply from want of attention to what are often considered trifling details, but which trifling details form, in my opinion, the most important portion of the treatment. By their use I believe that the majority of cases may be saved from the dreadful alternative of the loss of the limb, which, to the poor, whose living must depend on their daily labor, is of infinite importance, far more so than to those whose means place them beyond the necessity for exertion. It has been said that such cases are daily occurring, and occurring in every one's practice. Admitted: but does not this prove that the opinions expressed are incorrect, and if these and others have had the use of their limbs restored, there are, in all probability, hundreds who have suffered amputation that might have possessed a useful member? It is an easy matter to take off a leg, but the consequences to the patient exist for life. How often is it said, in similar cases, 'we must first get the patient into better health, and then perform amputation.' Now I want to know how a patient suffering from this disease in its most severe form can, during the existence of that disease, and in spite of it, be brought into better health, into such a state of health as will admit of his bearing the operation, and yet the health should not continue to improve, and with proper attention the disease should not be cured also. It is certain, if the severe symptoms I have mentioned be allowed to continue, death must be the result; but until you can prove to me that the disease is, from its nature, incurable, (I mean, of course, in young subjects) I am not prepared to admit either the necessity of amputation, nor that the disease will continue and destroy life, unless that extreme measure is resorted to. Again, supposing the bones themselves diseased, and the integrity of the joint destroyed, what is to prevent the restorative process from going on, and a comparatively useful limb being secured to the patient? We have evidence in abundance of this being effected in the instances of ankylosed joints that present themselves almost daily, of the hip, knee, and elbow. The misfortune is, and what I would particularly draw attention to, that from the comparatively low organized condition of the structure of the joint, the efforts of restoration are proportionably slow, and occupy many months of treatment, and of course, of attention; but what is this compared with the life of the individual, and his being enabled to obtain his subsistence in any capacity he may be placed? whereas, with a wooden leg, no very active employment can be undertaken, for, independently of the appearances it presents, objections naturally arise to the employment of such individuals. My object in making these remarks is solely to direct attention to what appears to me a very much neglected disease, and I believe, in many instances, this arises from an impression that it is a disease, if not incurable, at least so far hopeless that it is useless to waste the health or time of the patients in any attempts at cure.

If in these remarks I shall be the means of drawing attention to the subject, and in this way of saving the limbs of patients thus afflicted, whatever may be the opinion entertained, I shall have obtained more than I have any reason in right to expect. The assertion of these views is a bold step, and I am well aware that my motives may and will be questioned, and my opinion disputed. I have, however, a duty to perform in common with all men, and I trust never to be deterred by fear from openly and decidedly stating my opinions, believing conscientiously that they are correct, and believing also that general good may result from them."

We think the above cases and quotations will sustain our assertion, that scrofulous disease of the larger articulations is one of those in which limbs may be saved, which are ordinarily condemned to amputation.

Whether or not this is the case, the facts and views will serve to exhibit in a more striking point of view than could otherwise be done, the culpability of those who resort to amputation in such cases before suppuration, or any alarming symptoms have taken place. This is occasionally done in this region, under circumstances where the opinion of an enlightened medical public cannot operate with sufficient force to prevent it.

Several cases of the kind have come within our knowledge of which it may be sufficient at present to specify a single one.

December 6, 1845, we were requested to visit W. M., a young man of about 20 years of age, residing 23 miles south from this place, for the purpose of amputating his thigh. We visited him in company with our colleague, Prof. Herrick, and found him in the following state: the right knee was somewhat swollen, red, tender, and painful without any sign of suppuration.

Slight irritation of the general system, but no chills, sweats, or diarrhea. The disease had existed for several years, but so slightly as to allow him to follow some useful employment, and had recently become aggravated while he was undergoing a course of active medication. Of course we declined performing amputation, and advising gentle alteratives, evaporating lotions to the part, with anodynes if the pain was severe, encouraged him to hope that by perseverance, he would preserve not only his life, but a useful limb.

Soon after we learned that amputation had been performed.

We think it but just that the utmost publicity should be given to this case, and may have occasion to add others of a similar character, in order that those who prefer the triumph of an amputation to the credit of preserving a useful member, may receive the full benefit to be derived from a practical application of their principles.

