# Erratum

**Published:** 1846-06

**Authors:** 


ERRATUM.—Page 115, 4th line from top, for *right* read *left*.

